# Optimization of 3D-aggregated spheroid model (3D-ASM) for selecting high efficacy drugs

**DOI:** 10.1038/s41598-022-23474-5

**Published:** 2022-11-07

**Authors:** Sang-Yun Lee, Hyun Ju Hwang, Dong Woo Lee

**Affiliations:** 1 Department of Biomedical Engineering, Gachon University, Seongnam, 13120 Republic of Korea; 2 Central R&D Center, Medical & Bio Decision (MBD) Co., Ltd, Suwon, 16229 Republic of Korea

**Keywords:** Drug discovery, Drug screening

## Abstract

Various three-dimensional (3D) cell culture methods have been developed to implement tumor models similar to in vivo. However, the conventional 3D cell culture method has limitations such as difficulty in using an extracellular matrix (ECM), low experimental reproducibility, complex 3D cell culture protocol, and difficulty in applying to high array plates such as 96- or 384-plates. Therefore, detailed protocols related to robust 3D-aggregated spheroid model (3D-ASM) production were optimized and proposed. A specially designed wet chamber was used to implement 3D-ASM using the hepatocellular carcinoma (HCC) cell lines, and the conditions were established for the icing step to aggregate the cells in one place and optimized ECM gelation step. Immunofluorescence (IF) staining is mainly used to simultaneously analyze drug efficacy and changes in drug-target biomarkers. By applying the IF staining method to the 3D-ASM model, confocal microscopy imaging and 3D deconvolution image analysis were also successfully performed. Through a comparative study of drug response with conventional 2D-high throughput screening (HTS), the 3D-HTS showed a more comprehensive range of drug efficacy analyses for HCC cell lines and enabled selective drug efficacy analysis for the FDA-approved drug sorafenib. This suggests that increased drug resistance under 3D-HTS conditions does not reduce the analytical discrimination of drug efficacy, also drug efficacy can be analyzed more selectively compared to the conventional 2D-HTS assay. Therefore, the 3D-HTS-based drug efficacy analysis method using an automated 3D-cell spotter/scanner, 384-pillar plate/wet chamber, and the proposed 3D-ASM fabrication protocol is a very suitable platform for analyzing target drug efficacy in HCC cells.

## Introduction

Cell-based drug screening is crucial in the in vitro drug discovery process. Over the past few decades, the majority of high-throughput screening (HTS) has been performed on 2D monolayers. However, it is pointed out that the flat plastic bottom of the 2D monolayer cannot reflect various physiological relevance, so it is far from the in vivo environment^1^. To overcome these limitations, efforts to introduce a 3D-HTS method using spheroids are ongoing^2,3^. Till now, 3D-HTS has mainly been performed in matrix-free spheroids, such as hanging drop or ultra-low attachment (ULA) methods^4,5^. These methods show low throughput and low reproducibility due to the difficulty of changing clean culture media and the problem of spheroid loss. There is also a method for aggregating and culturing cells in 3D using mesh-type structures such as aggrewell^6^ and matrix-free microwell systems^7^. However, this method is a scaffold-free type and aggregated cells in media easily moved in media. They did not aggregate cells in the extracellular matrix (ECM). Therefore, these models lack physiological relevance due to the lack of ECM. Moreover, it has been difficult to apply to integrated array-based drug screening studies such as 96 or 384 plates due to the moving aggregated cells. To solve this problem, a 3D matrix-embedded culture method that can fix spheroids and create an extracellular matrix (ECM) environment using a bio-hydrogel such as Matrigel was introduced^8–10^. Matrigel is the most widely used 3D hydrogel and has important advantages for reflecting biological features, such as gene and protein expression, morphology, and cell–matrix interaction, still it also presents hurdles in its handling^11–13^. The greatest challenges are temperature sensitivity and high cost. These complex hurdles make it difficult to implement 3D-HTS models. To overcome these problems of 3D-HTS, our group developed a pillar chip plate that can form large-scale 3D spheroids using a small amount of bio-hydrogel in one plate^14–16^. In addition, in the pillar plate, because the spheroid is stably suspended at the end of the pillar tip by bonding with the hydrogel, fresh media change and drug screening are possible with only one stamping, thereby offsetting the need for labor-consuming pipetting. Our research team has previously reported various 3D-HTS analysis data using pillar plates and micropillar/well chips^17–22^. In particular, an aggregated spheroid model (ASM) that maximized drug efficacy evaluation by recapitulating the tumor microenvironment was attempted and reported in hepatocellular carcinoma (HCC)^23^. Therefore, pillar plate-based 3D-ASM has the advantage of being able to mix ECM with cells as a scaffold-type 3D cell culture method, and stable 3D cell culture and research with high uniformity and reproducibility.

As such, 3D-ASM-based HCC studies can be valuable tumor-mimicking models. This paper describes a detailed spheroid culture protocol and experimental optimization procedure that can generate more robust and stable 3D-ASMs. We uniformly dispensed the two HCC cell lines, Hep3B and HepG2,-Matrigel, onto a pillar plate using an automated spotter. After collecting the spotted cells using gravity, the humidity and temperature timing for gelation of Matrigel on the pillar were established and optimized to form 3D-ASM stably. In addition, immunofluorescence (IF) staining is often used to precisely analyze drug efficacy and changes in target biomarkers^24–27^. To implement this, immunochemical staining was performed using 3D-ASM, and confocal microscopic imaging, and 3D deconvolution image implementation and analysis were performed. Finally, the difference in drug sensitivity between the conventional 2D-HTS and 3D-ASM HTS was compared by analyzing the obtained HTS data, and the high drug efficacy against sorafenib, an HCC-approved drug^28–30^, in 3D-ASM HTS was verified. This report highlights the good drug efficacy analysis performance of the 3D-HTS platform based on an automated cell divider, 384-pillar plate, robust 3D-ASM implementation method, and 3D deconvolution image analysis using 3D confocal images.

## Materials and methods

### Cell Preparation and Culture

All human HCC cell lines (Hep3B and HepG2) were purchased from the Korean Cell Line Bank (Seoul, South Korea). Cells were grown in the Dulbecco′s Modified Eagle′s Medium (DMEM) medium (Gibco, Grand Island, NY) with 100 µg/mL of streptomycin, 100 units/mL of penicillin, 250 ng/mL of amphotericin B, and 10% fetal bovine serum. 10,000 units/mL Penicillin, and 10,000 (μg/mL) Streptomycin stock solutions are used for reducing the chances of microbial contamination in cell culture. Between 0.5 and 1 mL of Penicillin–Streptomycin solution are added to 100 mL of cell culture media for a final concentration of 50 to 100 units/mL penicillin and 50 to 100 μg/mL streptomycin. The cell lines were maintained at 37 °C in a 5% Carbon dioxide (CO_2_)-humidified atmosphere and routinely passaged every 4 days at 70% confluence. We used all cell lines under 20 passages after thawing the frozen cell stock. A frozen stock was established immediately after receiving each cell line, and only early passage (< 2 months) cells from the initially established frozen cell lines were used in the study.

### Experimental Procedure Of 3d-Hts

**Figure 1:**
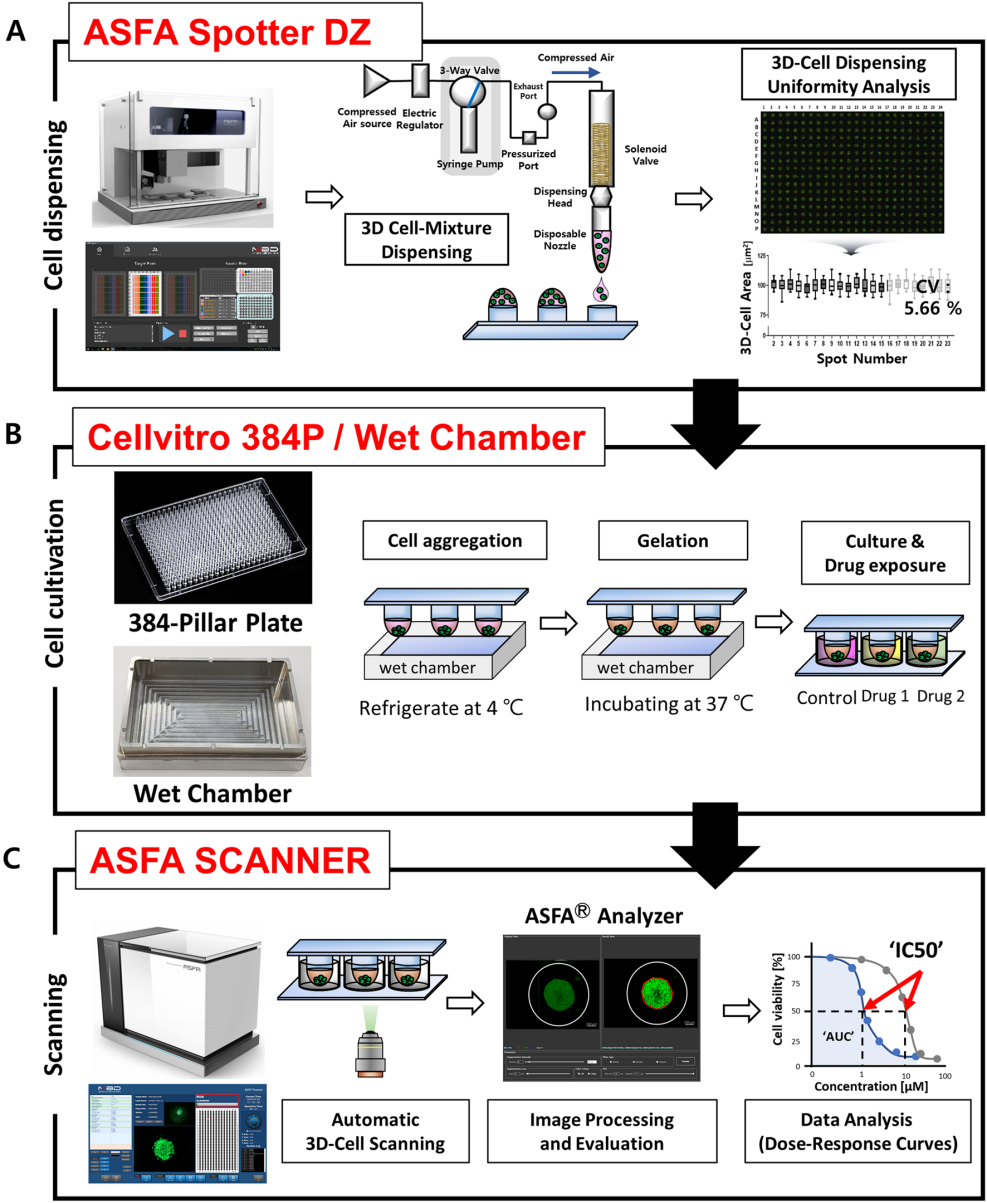
Schematic view of the 3D-Cell culture based high-throughput screening (HTS) platform for drug efficacy analysis in hepatocellular carcinoma (HCC) cells. (**A**) Schematic diagram of the sample dispensing and dispensing uniformity performance evaluation process using the proposed 3D-Cell spotter. (**B**). 384-Pillar plate photo and experimental procedure for 3D cell culture and drug efficacy test. (**C**). Quantitative analysis of drug efficacy using an automated optical scanner and image analysis software.

For HTS experiments, a 3D-cell spotter that automatically dispenses the samples is required. Therefore, we used an ASFA Spotter DZ (AS Fast and Accuracy spotter with disposable nozzles, Medical & Bio Decision, South Korea). This automatic 3D-cell spotter can dispense any desired volume of cancer cell-hydrogel mixture onto target plates at nL to uL levels. The manufactured and proposed ASFA Spotter DZ comprises an electric regulator, syringe pump, dispensing head, and disposable nozzles. This ASFA Spotter DZ quantitatively dispenses a liquid sample by controlling the pressure generated from the compressed air source using an electric regulator. When the experimenter loaded the sample into the source plate, the syringe pump created a negative pressure to aspirate the sample through the disposable nozzle and then automatically dispensed the sample to the target plate. Thus, the viscous 3D cell sample can be uniformly dispensed onto the target plate owing to the fine pressure control of the electric regulator, and the rapid sample aspiration process using the syringe pump. In addition, because the disposable nozzle was automatically replaced whenever the sample was changed, the washing step could be omitted in the middle of the experiment, establishing a very efficient process. To verify the dispensing performance of the ASFA Spotter DZ, it was quantitatively evaluated as a value of the coefficient of variation (CV) for uniform dispensing of cells mixed with the viscous hydrogel. The CV value analyzed based on the area value of the live cells dispensed to 384 individual spots was 5.66%, confirming high dispensing consistency (Fig. 1A).

The 384-pillar plate is made of polystyrene and contains 384-pillars (with a 2 mm pillar diameter and 4.5 mm pillar-to-pillar distance), which were manufactured by plastic injection molding. Plastic molding was performed using an injection molder (Sodic Plustech Inc., IL, USA). Therefore, 384-pillar/well plates are robust and flexible materials for mammalian cell culture, enzymatic reactions, viral infections, and compound screening. Using the proposed ASFA Spotter DZ, breast cancer cells were dispensed on a target plate and cultured for seven days to form multi-spheroids, and organoid culture was successfully performed. In this study, we successfully completed the icing step to aggregate the 3D-cells into one place by combining the wet chamber with the 384-pillar plate and the gelation step to fix the ECM Matrigel. The 384-pillar plate can be combined with a commercialized 384-well plate to incubate 3D cultured cells and the targeted cancer drug efficacy test **(**Fig. 1B**)**.

The 384-well plate was divided into twelve regions. Each region was composed of a 3 × 7 well array corresponding to six types of anticancer drugs in a two-fold and seven-point serial dilution series from 100 μM to 3.125 nM. (including one DMSO control) and in triplicate. 3D cultured HCC cells exposed to anticancer drugs were incubated at 37 °C in a 5% CO_2_ humidified incubator for 7 days. After incubation, 384 Pillar plate, in which HCC cells were cultured, were combined with a new 384 well plates containing live-cell staining solution, specifically staining only living cells after drug treatment. For live-cell staining, the staining solution was prepared by adding 1 µL of calcein AM to 7 mL of DMEM. Cells were incubated with staining solution for 1 h at 37 °C in a 5% CO_2_-humidified atmosphere. Live cell images with green fluorescence intensities (excitation/emission, 494/517 nm to lasers) were scanned using an optical scanner (ASFA Scanner, Medical and Bio Decision, Korea). Scanned images were evaluated using an image analysis software (ASFA Analyzer, Medical and Bio Decision, Korea). We performed a more specific and accurate drug response analysis using the dose–response curve (DRC) according to the concentration gradient (GraphPad Prism 9, GraphPad Software, CA). Based on the graph, the drug response to anticancer drugs was confirmed in individual cells through the quantified inhibitory concentration 50 (IC50) and area under curve (AUC) values (Fig. 1C).

### Cell Viability Assay For 2D-HTS

For 2D-HTS analysis, HCC cell lines were mixed with fresh cell culture medium containing no ECM, and then samples prepared for 2D cell culture at a concentration of 2000 cells per 40uL volume were manually dispensed using a pipette. One day after seeding, using an ASFA Spotter DZ, drugs were dispensed in the same drug layout and concentration gradient as the 3D-HTS experimental conditions on a 384 well plate for 2D-HTS. After six days of incubation at 37 °C in a 5% CO_2_ humidified incubator, cell viability was determined using the adenosine triphosphate (ATP) monitoring system based on firefly luciferase (CellTiter-Glo® Cell Viability Assay, Promega, Madison, WI), according to manufacturer’s protocol. The ATP assay mixture was prepared by adding 10 µL of CellTiter-Glo reagent to 40 µL of the medium per well. Cells were lysed with an assay mixture by shaking for 2 min, and luminescence was recorded 10 min after reagent addition using the SpectraMax iD3 Reader (Molecular Devices LLC, San Jose, CA).

### Optimized protocol of 3D-ASM preparation

**Figure 2:**
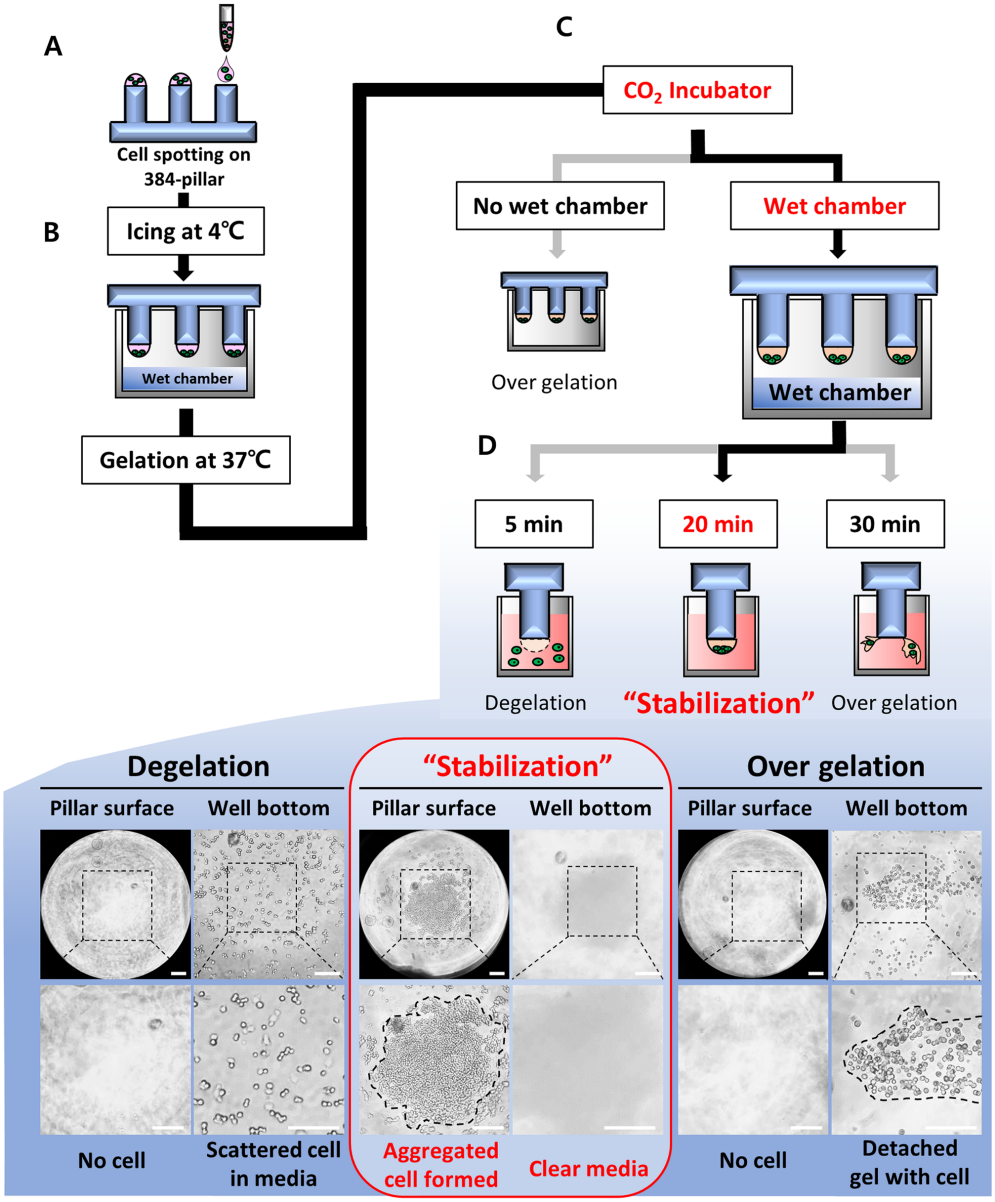
Optimized 3d cell culture protocol for implementing 3D-ASM. (**A**) Cell and matrigel mixture dispensed on the surface of the 384-pillar plate by 3D-Cell spotter. (**B**). The 384-pillar plate aggregates the cells into one place through storage in an ice box for 2 h while combined with the wet chamber. (**C**). In the CO_2_ incubator condition, over gelation occurs if the wet chamber and the 384-pillar plate are not combined. (**D**). In humidified incubator and wet chamber conditions, the 3D-cell mixture is stabilized after gelation for 20 min. (de-gelation occurs at the 5-min condition, and over gelation occurs at the 30-min condition).

**Figure 3:**
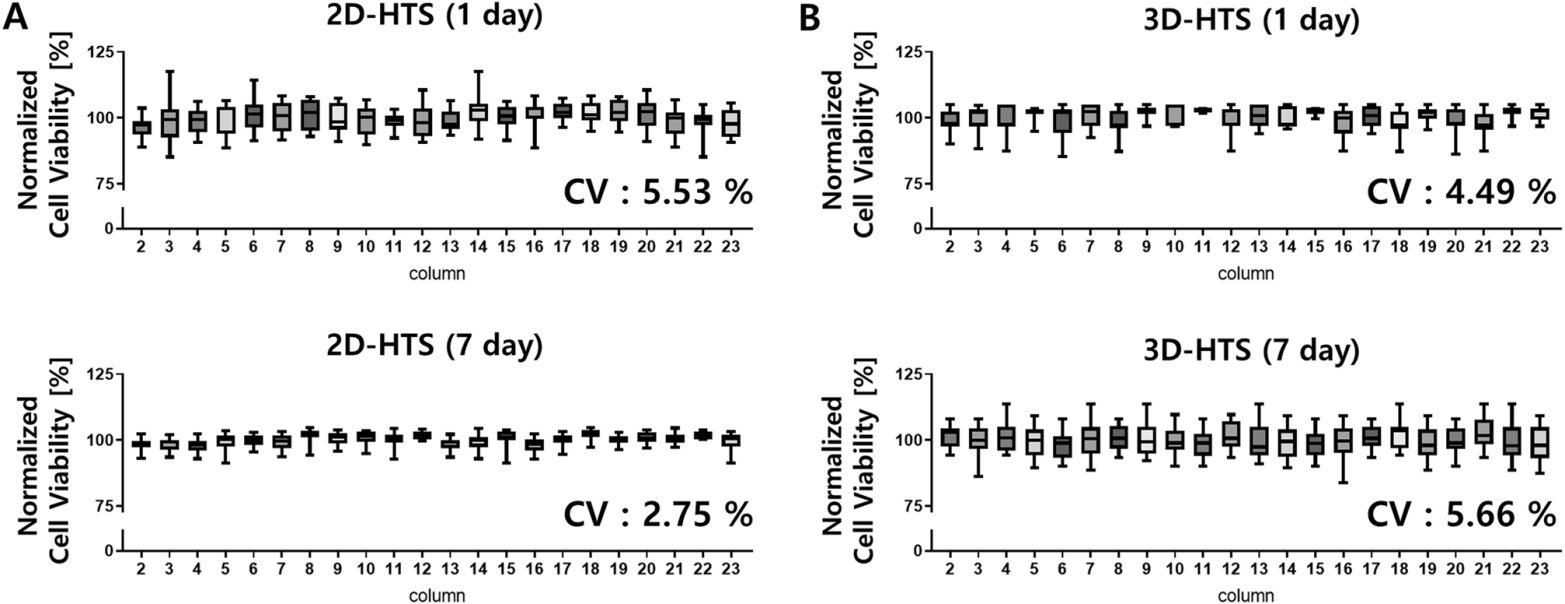
Quantitative analysis of cell dispensing and culture uniformity through cell viability measurement. (**A**) The uniformity CV values of the 1 and 7 days of culture after dispensing the cells in the 2D-HTS condition were 5.53% and 2.75%, respectively. (N = 308, spots excluding the edge of the 384 plate). (**B**). The uniformity CV values of the 1 and 7 days of culture after dispensing cells in 3D-HTS conditions were 4.49% and 5.66%, respectively. (N = 308, spots excluding the edge of the 384 plate).

To make 3D-ASM, dispense the cell-Matrigel mixture on the 384-pillar surface using an automated ASFA Spotter DZ, as shown in Fig. 2A. The 384-pillar plate to which the sample was dispensed was combined in a wet chamber and kept in an ice box maintained at 4 °C for 2 h. This process aggregated single cells in one place (Fig. 2B). To culture the cells aggregated into one place in 3D, the ECM, Matrigel, was hardened by temperature. Because Matrigel is very sensitive to temperature, so it hardens in a 37 °C incubator, which is called gelation. Even under CO_2_ incubator conditions, if the wet chamber and the 384-pillar plate are not connected, there is a problem that the 3D-cell-Matrigel mixture is over-gelated (Fig. 2C). Therefore, we established a stabilized 3D-ASM production condition by incubating a 384-pillar plate in a wet chamber for 20 min in a humidified incubator (Fig. 2D). To implement 3D-ASM on a 384-pillar plate, the proposed wet chamber must be used from the icing step (aggregating individual cells) to the gelation step. In addition, it is crucial to follow the exact temperature and time conditions, as suggested in the icing and gelation steps. Also, since Matrigel is a collagen-based bio-derived material, there may be a lot-to-lot variation issue^31^. Therefore, as shown in Fig. 2, the 3D-ASM produced according to the optimized protocol was imaged and observed, and to perform 3D-HTS analysis, the 3D cell viability was quantitatively measured to confirm whether the cells were uniformly cultured in 3D (Fig. 3).

### Confocal microscope image scanning and 3D-deconvolution image implementation

The 3D cultured HCC cells were fixed in 2% paraformaldehyde (PFA) for 40 min and further incubated with 0.25 mg/mL Sodium Borohydride (NaBH_4_) for an additional 40 min. The antibody staining solution was prepared by adding Hoechst 33,342 (1000:1, Thermo Fisher Scientific, Korea, H6024, blue fluorescent dye) and Alexa Fluor 488 phalloidin (1:1000, Invitrogen, Waltham, MA, USA, A12379, green fluorescent dye) to the permeabilizing and blocking solution. Samples were exposed to staining solution for 1 d at 4 °C. The stained 3D cells were washed for 15 min in staining buffer solution (MBD-STA500, Medical and Bio Decision, South Korea) before imaging to remove background fluorescence noise. A total of 30 confocal image sections were acquired at 5 μm intervals (confocal microscope system, ZEISS LSM 780, Carl Zeiss, Germany), and 3D-deconvolution images were created using ImageJ software^32,33^ (open-source Java-based image processing and analysis software, US National Institutes of Health, USA) to analyze the fluorescence intensity.

## Results

### Quantitative analysis of the 3D cell-forming uniformity in each 3D cell model

To quantify the drug response through HTS experiments, it is essential that target cells are uniformly dispensed and cultured. In general, when performing 96- or 384-well plate-based experiments, the experiment, and analysis are performed except for the edge in consideration of the ‘edge effect’ of drying the edge. Therefore, the X-axis in Fig. 3 means 22 columns (2 to 23) in the middle, excluding the columns at both ends of the 384-pillar plate. And a box plot for each column means the average and standard deviation values of the middle 14 rows (B ~ O) excluding the two end rows. Therefore, we evaluated the reproducibility of the HTS analysis using the coefficient of variation (CV) of 308 spots excluding the edge of the 384 plate. Generally, if a CV value is less than 10%, it is evaluated as a good cell-based assay^34–36^. For the 2D-HTS assay, cells were dispensed into a 384 well plate, and the CV value was measured. As a result, The CV values were calculated to be 5.53% on the 1 day of culture and 2.75% after 7 days of culture (Fig. 3A). In particular, because 3D-HTS is performed using 3D-ASM, it is very important to establish assay reproducibility based on CV values. In the case of 3D-HTS assay conditions, the CV value was confirmed to be 4.49% on the first day of 3D-ASM culture and 5.66% on the seventh day of culture (Fig. 3B). Therefore, the 3D-HTS assay performed using the 3D-ASM implementation method proposed in this study demonstrated a level of assay reproducibility similar to that of the conventional 2D-HTS assay. Therefore, the 3D-ASM implementation method proposed in this study has the advantage of implementing a unique 3D cell culture model, unlike the conventional 2D cell culture method. This is very useful because it shows high assay reproducibility and can be applied to 3D-HTS assays.

### Confocal image scanning and 3D deconvolution image analysis

**Figure 4:**
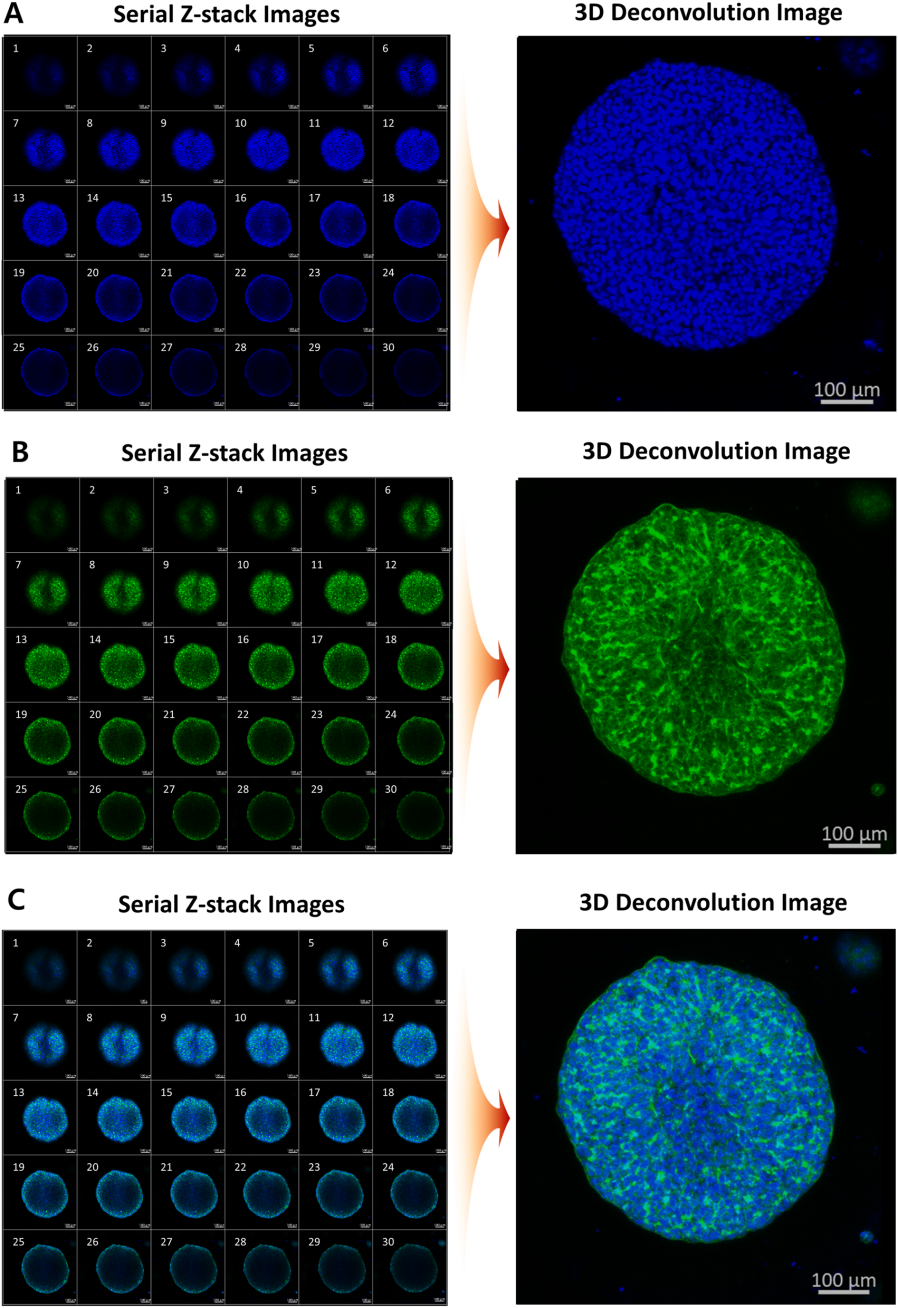
Confocal image scan and 3D deconvolution image implementation for 3D-ASM. (**A**) Serial Z-stack confocal imaging and 3d deconvolution image implementation after cell nucleus staining (blue). (**B**). Serial Z-stack confocal imaging and 3d deconvolution image implementation after cytoskeletal staining (green). (**C**). Implementation of serial Z-stack confocal image and 3d deconvolution image by merging cell nucleus and cytoskeleton images (blue and green merged).

To simultaneously analyze the quantitative drug efficacy and mechanism of action (MoA), the IF staining method capable of staining the cell nucleus, cytoskeleton, and target protein is mainly used. Therefore, we tested whether the experiment was possible without damaging the cell-Matrigel mixture, even when the IF staining experiment was performed using the proposed 3D-ASM. In addition, for 3D-cultured cells, image scanning using a confocal microscope and the realization of 3d deconvolution images are very important for accurate image analysis. IF staining requires numerous experimental steps such as cell fixation, blocking, permeabilization, and treatment with staining reagents. Therefore, we verified whether the proposed 3D-ASM structure was well maintained and stained successfully even after IF staining. After staining the cell nucleus (Fig. 4A) and cytoskeleton (Fig. 4B) of 3D-ASM, 30 individual images were scanned at 5 µm intervals using a confocal microscope. The staining reagents for staining cell nuclei and cytoskeleton were successfully stained inside the 3D-ASM and confirmed as a serial Z-stack image. A 3D deconvolution image was successfully implemented based on the scanned individual serial Z-stack images. In addition, as can be seen from the individual serial Z-stack images, in the case of 3D-ASM, it is very important to implement a 3d deconvolution image and then analyze the image because the scanned fluorescence area differs depending on the Z-axis position. Finally, serial Z-stack images were successfully obtained by merging individual images at different wavelengths, and 3d deconvolution images were obtained (Fig. 4C). As such, the 3D-ASM model proposed in this study enables target biomarker analysis based on IF staining and 3d deconvolution images. Therefore, it is possible to perform high content imaging (HCI) analysis that analyzes drug efficacy and drug MoA at the same time by accurately analyzing the intracellular expression location and expression level of target biomarkers.

### Comparison of hts drug efficacy analysis results according to cell culture models

**Figure 5:**
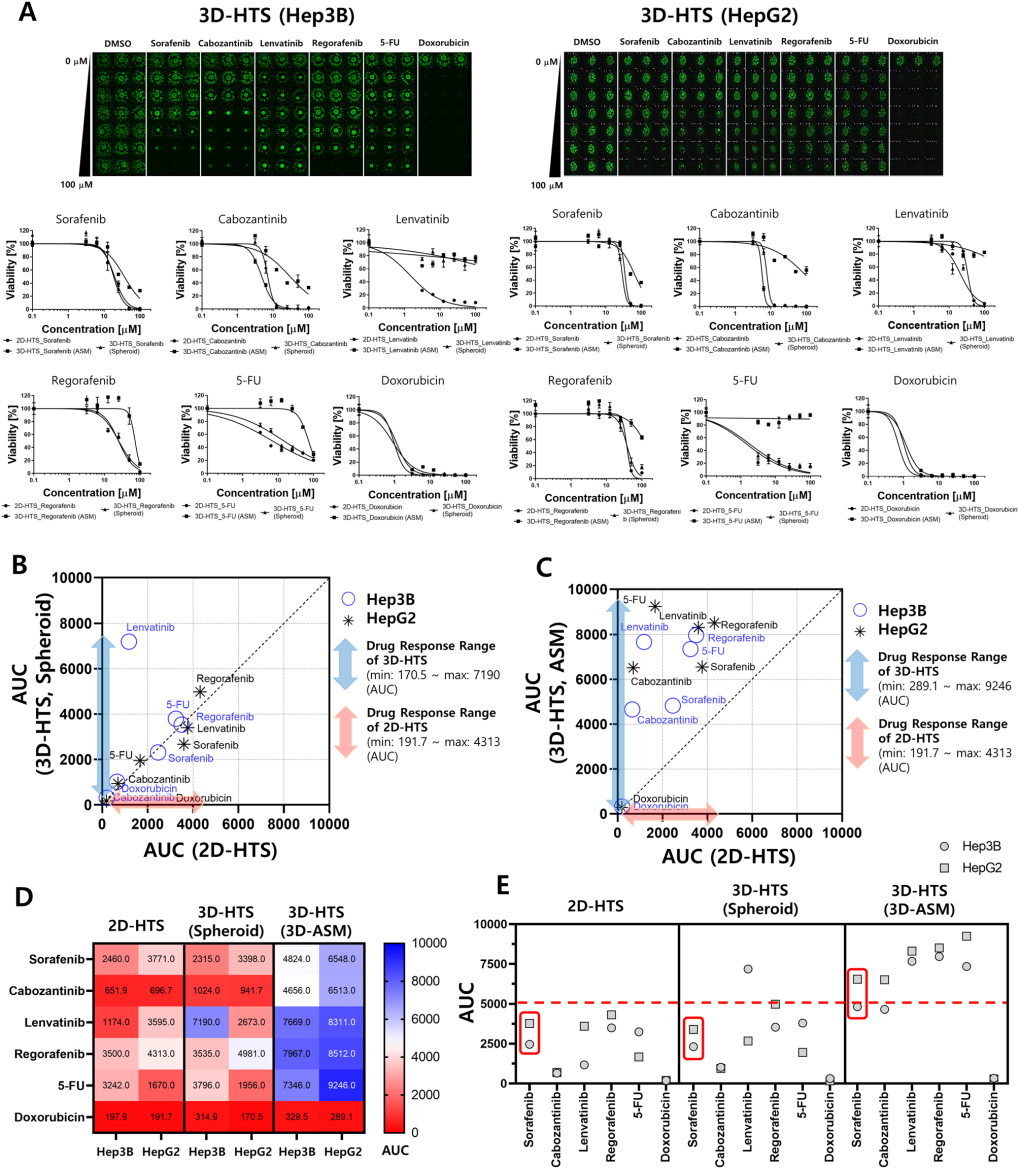
Confirmation of high drug efficacy analysis performance of 3D-HTS platform through comparison of HTS results. (**A**) Live cell images (3D-ASM). Dose–response curves (DRCs) of 2D-HTS and 3D-HTS (ASM and spheroid) by drug for two HCC cell lines (Hep3B and HepG2). (**B**). Comparative analysis of drug response according to AUC values based on 2D-HTS and 3D-HTS (spheroid and ASM). (**C**). Comparative analysis of drug response according to AUC values based on 2D-HTS and 3D-HTS (spheroid and ASM). (**D**). Comparative heatmap analysis of drug response based on AUC value. E. Comparative analysis of drug response based on AUC values according to the drug screening model. 2D-HTS and 3D-HTS (spheroid) showed a sensitive response to all drugs, and thus had low selectivity to the sorafenib.

Drug sensitivities to six anticancer drugs were compared using two HCC cell lines, under 2D-HTS and 3D-HTS conditions. For the 3D-HTS assay, two types of HCC cell lines were cultured in the previously proposed 3D-ASM form, after which drug screening was performed. As a result of the 3D-HTS assay, drug efficacy was quantified as the Area Under Curve (AUC) values based on live cell images and DRCs for the six individual anticancer drugs (Fig. 5A). Comparing AUC values, which are drug response analysis values of 2D-HTS 3D-HTS (Spheroid) and 3D-HTS (3D-ASM), most drugs showed resistance under 3D-HTS conditions (Fig. 5B,C). The blue and red double-headed arrows indicate the drug response measurement ranges of 3D-HTS and 2D-HTS, respectively. Interestingly, it can be seen that the drug response measurement range of 3D-HTS (3D-ASM) is wider than that of 2D-HTS. Also, 3D-HTS under conventional 3D-spheroid conditions had a wider range of drug reactivity than 2D-HTS, but narrower than 3D-HTS based on 3D-ASM. In addition, at heatmap analysis, 2D-HTS and conventional 3D-spheroid based 3D-HTS drug response analysis results mostly showed a red area of less than 50% level (AUC), whereas 3D-ASM based 3D-HTS drug response analysis results showed a much wider analysis area from about 0 to 100% level (AUC) (Fig. 5D). This not only means that drug resistance is higher in the 3D-ASM based 3D-HTS assay condition, but also suggests that the 2D-HTS and conventional 3D-spheroid based 3D-HTS assay is too sensitive to evaluate the efficacy of an effective drug in more detail. As a result of analyzing drug responses to six types of anticancer drugs, it was found that 2D-HTS was at a drug response level of less than 50% for all six drugs. Therefore, the sensitivity of the FDA-approved sorafenib drug for HCC was not high in 2D HTS assay. In contrast, in the 3D-ASM based 3D-HTS condition, the efficacy of FDA-approved sorafenib show higher efficacy than other drugs (except Doxorubicin). These results show that proposed 3D-ASM based 3D-HTS has better analytical discrimination than 2D-HTS and conventional 3D-spheroid based 3D-HTS (Fig. 5E). Therefore, in general, increased drug resistance under proposed 3D-ASM based 3D-HTS conditions does not reduce the analytical discrimination of drug efficacy, but rather improves the analytical performance of discriminating high-potency drugs compared to the conventional 2D-HTS assay.

## Discussion

There are various existing methods for culturing cells in 3D. The scaffold-free method that does not use an ECM allows cells to grow together in 3D using a hanging drop culture method^37,38^ or ULA plate of U/V bottom type^39,40^. In the Scaffold method, cells are cultured in 3D by mixing various ECMs such as collagen^41,42^ and Matrigel^43,44^ with the cells. In this case, a special culture vessel such as a well plate for 3D cell culture^45–47^ or a microfluidics chip^48,49^ is required. However, the 3D cell culture method has not yet been standardized, and manual dispensing of the cell/ECM mixture into 96 and 384 well plates is very inefficient and labor-consuming, so it is difficult to implement 3D cultured cells reproducibly. To overcome these drawbacks, in this study, we proposed a detailed protocol for culturing HCC cell lines in 3D-ASM format and successfully confirmed the ability to discriminate HCC cell lines with higher drug sensitivity under the existing 3D-HTS assay conditions (Fig. 1). Also, the 3D-cell spotter (ASFA Spotter DZ) makes it easy to dispensing cell/ECM mixtures with high uniformity compared to pipette dispenser. This has the advantage of securing high experimental reproducibility by simplifying user-friendly and labor-intensive experimental steps. The matrigel used as the ECM in this study is sensitive to temperature and hardens easily at room temperature^50^. To implement 3D-ASM, it was suggested that a wet chamber must be additionally used along with the icing process under low-temperature conditions and the gelation process in a humidified incubator by observing the suggested temperature and time (Fig. 2). In this paper, a method for stably producing 3D-ASM is presented. To determine a robust and reproducible assay, a means CV value of less than 10% is used for a good cell-based assay. The proposed 3D-ASM showed uniform CV values (Fig. 3) applicable to HTS studies. However, since each cell shows various growth rates and characteristics, it is necessary to test the number of cells in advance to implement 3D-ASM according to the user's experimental purpose and experimental conditions. Immunochemical staining and confocal imaging assays typically use fluorescence imaging of samples and report quantitative parameters such as spatial distribution of targets and individual cell and organelle morphology, which can provide a more systematic and accurate assessment of drug candidates^51,52^. Therefore, their application in this study is very important as these methods are integrated into all aspects of drug discovery in the pharmaceutical industry and scientific research in academia^53^. Therefore, 3D-ASM was confirmed to be applicable not only to immunochemical staining, but also to confocal microscope scanning, 3d deconvolution image implementation, and image analysis studies (Fig. 4). Using the implemented 3D-ASM, 3D-HTS assay, and conventional 2D-HTS assay were performed. Generally, 3D-organoids induce necrosis-like apoptosis in the core when cultured for a long period of time. Therefore, in this study, 3D cell culture and drug screening analysis were performed for a short period of 7 days. In 3D-cultured cells, the composition of the extracellular matrix, along with its physical properties, can influence the response of cells to drugs by altering the action mechanism of the drug or promoting drug resistance^54,55^. It has also been reported that when cancer cells are cultured in 3D, the expression of tumor-associated proteins is better maintained than that under conventional 2D cell culture conditions^56,57^. For these reasons, drug resistance is usually high in 3D-HTS conditions. Therefore, despite the increased drug resistance in 3D-HTS conditions, we wanted to confirm higher drug reactivity assay performance. Sensitivity to six anticancer drugs was quantitatively analyzed, and as a result, the drug sensitivity analysis area was wider under 3D ASM based 3D-HTS conditions, and the high drug efficacy analysis ability of the FDA-approved sorafenib drug for HCC was verified. In addition, it was confirmed that the drug sensitivity analysis area under the 3D-ASM condition was wider compared to the conventional 3D spheroid-based 3D-HTS assay result. In other words, these results indicate that increased drug resistance under 3D-HTS conditions does not reduce the analytical discrimination of drug efficacy, but improves the analytical performance of discriminating effective drugs compared to the conventional 2D-HTS assay (Fig. 5).

## Conclusion

This study demonstrated that the proposed 3D-ASM based 3D-HTS assay can be more appropriately applied to HCC-related drug development and research. In particular, an optimized protocol that can be applied to in vitro experiments by stably and reproducibly manufacturing 3D-ASM using the 384-pillar plate proposed by this research was presented. In the future, we plan to conduct research to identify drug MoA by examining the activation and inactivation of other drug-related pathways and genomes through molecular assays such as PCR analysis for drugs with different reactivity depending on cell culture models. Ultimately, it could be applied to comprehensive drug efficacy analysis research through the discovery of new drugs using patient-derived HCC cells and comparison with genome analysis.

## Data Availability

All data generated or analysed during this study are included in this published article.
