# Author Correction: Optimization of 3D-aggregated spheroid model (3D-ASM) for selecting high efficacy drugs

**DOI:** 10.1038/s41598-023-32198-z

**Published:** 2023-04-04

**Authors:** Sang‑Yun Lee, Hyun Ju Hwang, Dong Woo Lee

**Affiliations:** 1 Department of Biomedical Engineering, Gachon University, Seongnam, 13120 Republic of Korea; 2 Central R&D Center, Medical & Bio Decision (MBD) Co., Ltd, Suwon, 16229 Republic of Korea

Correction to: *Scientific Reports* 10.1038/s41598-022-23474-5, published online 07 November 2022

The original version of this Article omitted an affiliation for Sang-Yun Lee. The correct affiliations are listed below.

Department of Biomedical Engineering, Gachon University, Seongnam 13120, Republic of Korea.

Central R&D Center, Medical & Bio Decision (MBD) Co., Ltd, Suwon 16229, Republic of Korea.

Additionally, Dong Woo Lee was incorrectly affiliated with ‘Central R&D Center, Medical & Bio Decision (MBD) Co., Ltd, Suwon 16229, Republic of Korea.’ The correct affiliation is listed below.

Department of Biomedical Engineering, Gachon University, Seongnam 13120, Republic of Korea.

The original Article has been corrected.

